# Impact of maternal depression trajectories on offspring socioemotional competences at age 11: 2004 Pelotas Birth Cohort

**DOI:** 10.1016/j.jad.2019.03.072

**Published:** 2019-06-15

**Authors:** Jessica Mayumi Maruyama, Maria Pastor-Valero, Iná S. Santos, Tiago N. Munhoz, Fernando C. Barros, Alicia Matijasevich

**Affiliations:** a Departamento de Medicina Preventiva, Faculdade de Medicina FMUSP, Universidade de Sao Paulo, Av. Dr. Arnaldo, 455, 2nd floor, Sao Paulo, SP, CEP 01246-903, Brazil; b Department of Public Health, History of Science and Gynecology, Faculty of Medicine, Miguel Hernández University, Campus de San Juan, Spain; c CIBER in Epidemiology and Public Health (CIBERESP), Madrid, Spain; d Postgraduate Program in Epidemiology, Federal University of Pelotas, Pelotas, Brazil; e Faculty of Psychology, Federal University of Pelotas, Pelotas, Brazil; f Postgraduate Program in Health and Behaviour, Catholic University of Pelotas, Pelotas, Brazil

**Keywords:** Maternal depression, Socioemotional competence, Adolescent, Cohort study

## Abstract

**Background:**

Maternal depression is associated with impairments in child behavioural and emotional development, although the effect of exposure to maternal depression until adolescence is underexplored in most studies. This longitudinal study examined the association between maternal depressive symptoms trajectories and offspring socioemotional competences at age 11.

**Methods:**

We included 3,437 11-year-old adolescents from the 2004 Pelotas Birth Cohort Study. Maternal depressive symptoms were assessed during the follow-up waves. Adolescent socioemotional competences were peer relationship problems and prosocial behaviour, both assessed by Strengths and Difficulties Questionnaire (SDQ), and Locus of Control (LoC), assessed by Nowick-Strickland Internal-External Scale. We used multivariate linear and logistic regression models to examine the effects of maternal depression trajectories on offspring's socioemotional competences, adjusting for potential confounding variables.

**Results:**

We identified five trajectories of maternal depressive symptoms: a “low” trajectory (32.6%), a “moderate low” (42.2%), a “increasing” (11.1%), a “decreasing” (9.2%), and a “high-chronic” trajectory (4.9%). Adolescents whose mothers had persistent depressive symptoms, either intermediate or high, had greater levels of peer relationship problems and lower levels of prosocial behaviour than those whose mothers had low depressive symptoms. These differences were not explained by socioeconomic, maternal, and child characteristics. Maternal depressive symptoms during offspring's life was not a predictor of LoC orientation.

**Limitations:**

Nearly 20% of original cohort were not included in the analysis due to missing data. Adolescent's socioemotional competences were ascertained by maternal report.

**Conclusion:**

Our study extended the evidences of the negative impact of severe and recurrent maternal depression on offspring's socioemotional competences until early adolescence.

## Highlights

• Persistent maternal depression after birth was associated with offspring´s socioemotional difficulties at age 11.
• Adolescents from persistent depressive mothers had high levels of peer relationship problems.
• Low levels of prosocial behaviour were also associated with persistent maternal depression.
• These associations were not explained by socioeconomic, family, and child characteristics.
• Maternal depression during adolescent's life was not a predictor of locus of control orientation.

## 1 Introduction

Maternal depression after childbirth is a common psychiatric disorder, affecting between 10 to nearly 20% of women in high- and low- or middle-income countries, respectively (Fisher et al., 2012, Howard et al., 2014). It is associated with a variety of individual and contextual risk factors, such as previous depressive episode, low socioeconomic status, lack of social support, marital difficulties and single-parent household (Ashman et al., 2008, Goodman et al., 2011). In vulnerable women, pregnancy complications and offspring's health and behaviour problems may lead to depressive symptoms (Howell et al., 2009).

Besides the deleterious consequences for the woman herself, maternal depression is also associated with impaired mother-infant relationship and adverse child development outcomes. Compared to controls with no psychiatric disorders, depressed mothers presented greater difficulties in parental behaviour, including harsher and more negative interactions and unpredictable or inconsistent response to offspring's needs (Lovejoy et al., 2000). Numerous evidences showed that maternal depression is a risk factor for the development of offspring psychopathology and other negative outcomes, including delayed cognitive and language development, higher rates of mood disorders, such as depression, and internalizing and externalizing problems (Grace et al., 2003, Goodman, 2007, Hammen, 2009, Sanger et al., 2015). In addition, according to psychosocial developmental perspective, maternal depression is also associated with a variety of poor social and emotional functioning symptoms in youth, with deleterious consequences to long-term well-being of individuals (Goodman et al., 2011, Klein et al., 2009).

In recent years, some studies evaluated not only severity or timing, but also the persistence of maternal depression and its impact on a variety of child socioemotional and behaviour outcomes (Ahun et al., 2017, Ashman et al., 2008, Campbell et al., 2007, Cents et al., 2013, Giallo et al., 2015, Guyon-Harris et al., 2015, Kingston et al., 2018, Matijasevich et al., 2015, Netsi et al., 2018, Park et al., 2018, Raskin et al., 2016, Van der Waerden et al., 2015). In general, these studies identified two to six maternal postnatal depression trajectories, from none or minimal depressive symptoms trajectories to high chronic symptoms trajectories. Also, they mostly reported that children from mothers belonging to persistent and severe depressive symptoms trajectories were at more risk to present emotional and behavioural difficulties when compared to those from mothers with minimal or low depressive symptomatology, even after adjusting for potential confounding variables.

Adolescence represents a particularly at-risk stage for the onset of several mental disorders and risk behaviours, notably conduct problems and substance abuse, which are strongly associated with higher risk of criminality and incarceration in adulthood (de Girolamo et al., 2012). This period of life is marked by social changes that results in more autonomy and more influence and time spent with peers rather than parents. Several studies showed that a high quality parent-adolescent relationship is a protective factor against socioemotional adjustment difficulties and substance abuse disorders (Hardaway et al., 2012, Steinberg, 2001). Wherefore, maternal depression may exert negative effects on youth's socioemotional competences as a consequence to the difficulties in the relationship with a depressed mother on the transition from childhood to adolescence.

The majority of the studies regarding the repercussion of maternal depression trajectories on offspring's behavioural or emotional problems were focused on early to late childhood and their mainly measured outcomes were psychopathologies symptoms. There is a lack of information concerning the consequences of exposure to maternal depression until adolescence, especially focusing on socioemotional competences (Sanger et al., 2015) Also, there is scarce evidence from low- and middle-income countries, since almost all of the studies used data from high-income countries, such as USA, Canada and France, which may present relevant differences in social and cultural contexts (Berger et al., 2016, Howard et al., 2014). Therefore, the current study, conducted in a middle-income country, aimed to evaluate the impact of maternal depression trajectories on offspring's three socioemotional competences (peer relationship problems, prosocial behaviour and locus of control) at age 11 accounting for numerous potential confounders.

## 2 Method

### 2.1 Participants

The 2004 Pelotas Birth Cohort is a population-based birth cohort of children born in the city of Pelotas, Southern of Brazil, from Jan 1, 2004 to Dec 31, 2004. All hospital births throughout that year were identified during daily visits to the city's five maternity hospitals (over 99% of deliveries took place in hospitals) (Santos et al., 2011). There were recruited 4231 live births of mothers living in the urban area of Pelotas (non-response rate at recruitment was < 1%). The detailed methodology was described elsewhere (Santos et al., 2011, 2014). Mothers were interviewed and their children examined within the first 24 h after birth. A structured questionnaire was administered to collect information on demographic, socioeconomic, biological and behavioural characteristics. Follow-up assessments were made at home at mean (standard deviation) ages 3.0 (0.1), 11.9 (0.2), 23.9 (0.4) and 49.5 (1.7) months and at a research clinic at 6.8 (0.3) and 11.0 (0.3) years, with follow-up rates between 87 and 96%.

### 2.2 Main exposure: maternal depressive symptoms

Maternal depressive symptoms were assessed by the Edinburgh Postnatal Depression Scale (EPDS) (Cox et al., 1987). The EPDS is a self-report, 10-item scale (score range: 0–30, with higher scores indicating more severe depressive symptoms) that expresses the intensity of depressive symptoms over the preceding seven days. We used a previously translated and validated version of the questionnaire (Santos et al., 2007). The EPDS was administered to almost all mothers at each follow-up, except at the 3-month follow-up when it was completed by a subsample of 965 mothers.

The trajectories of maternal depression symptoms were constructed using EPDS scores from 3 months to 11-year follow-up through a semi-parametric group-based modelling approach (Nagin, 2005, Nagin and Tremblay, 1999). Group-based trajectory modelling is a specialized form of finite mixture modelling. Details of the steps and methods used to identify the trajectories of maternal depressive symptoms were reported in previous studies (Azeredo et al., 2017, Matijasevich et al., 2015). Briefly, 90% of the sample population completed the EPDS at least three times and 17% of mothers completed the EPDS in all follow-ups. We included 3841 mothers with data from at least three follow-ups in the analyses. Individuals with missing information were not excluded from the model due to the ability of group-based trajectory modelling to handle missing data using maximum likelihood estimation (Nagin, 2005). The number and shape of trajectories were based on the best fit of the model (maximum Bayesian information criteria, BIC) and on the interpretability of the trajectories obtained (Nagin, 2005). In order to model trajectories of maternal EPDS scores, analyses were conducted specifying three-, four-, five-, and six-group models. BIC improved as more groups were added. The improvement observed when moving from the five-group to the six-group model was low and the five-group model emerged as the best fitting and most parsimonious model. In addition, selection of the appropriate model was guided by the posterior probability scores for each trajectory group (i.e., the individual's probability of belonging to each of the trajectory groups). For all five groups, the average posterior probability was above the lower recommended threshold for assignment of 0.7 (average posterior probability of 0.87, 0.81, 0.78, 0.79 and 0.87 for Group 1 to Group 5, respectively) (Nagin, 2005). Inspection of parameter estimates for the five-group model revealed that the constant term differed from zero for all five groups.

### 2.3 Main outcomes: adolescent's socioemotional competences

The present study evaluated three different socioemotional competences of adolescents at age 11: peer relationship problems, prosocial behaviour and locus of control.

Peer relationship problems and prosocial behaviour were ascertained by the mothers or caregivers using subscales of the Strengths and Difficulties Questionnaire (SDQ) (Goodman, 1997). The SDQ peer relationship problems subscale consists in a 5-item questionnaire with score range from 0 to 10, with greater scores representing greater difficulties. The SDQ prosocial behaviour subscale is also a 5-item questionnaire with score range from 0 to 10, but greater scores represent lower difficulties. We analysed SDQ peer relationship problems and prosocial behaviour as continuous variables in original score units and also as binary outcomes, according to SDQ's scoring website (SDQ INFO, 2016). The adolescent was considered as having peer relationship problems if scored ≥ 4 points and as having prosocial behaviour problems if scored ≤ 4 points in the subscale. The instrument was validated for use in the Brazilian population by Fleitlich et al. (2000).

Locus of control (LoC) was assessed by the Nowick-Strickland Internal-External Scale (CNSIE) (Nowicki and Strickland, 1973). The shortened CNSIE version consists in a 12-item test using a "Yes-No" response format. A total score was derived by summing scores for all the items, with higher scores indicating a more external LoC. A person with a higher ‘internal’ score is considered to perceive that the outcome of events is under their own control, whilst a person with a higher ‘external’ score on this measure is considered to perceive that the outcome of events is the result of luck or random factors. The questionnaire has been shown to have good construct validity and test-retest reliability in children from ages 9 through 18years (Nowicki, 1976, Nowicki and Duke, 1974, Nowicki and Strickiland, 1973). The scale was translated to Portuguese and its psychometric properties were positively evaluated by Barros et al. (1992). For participant missing responses to one or two LoC items (e.g., less than 20% of the total scale items), unanswered questions were replaced with the mean of the participants' own responses to the rest of the scale items (Culpin et al., 2015). From a total of 3531 children, 132 (4,0%) had their unanswered responses replaced by their own scores' mean and 10 (0,3%) were excluded from analysis for presenting three or more missing responses.

### 2.4 Covariates

Information about maternal, pregnancy, family and child characteristics was gathered at the perinatal interview unless indicated otherwise. Socioeconomic and maternal characteristics were: monthly family income in the month prior to delivery (quintiles); maternal schooling (number of completed years of formal education, categorized as 0–4, 5–8, and ≥9 years); maternal self-reported skin colour (white or non-white); maternal age at childbirth (<19, 20–34, ≥35 years old); marital status (single mother or living with a partner); parity (the number of previous viable pregnancies resulting in a live birth or a late fetal death, categorized as 0, 1 or ≥2).

Smoking and alcohol use during pregnancy were self-reported and were evaluated retrospectively at birth. Women were categorized as having smoked during pregnancy if they reported smoking at least one cigarette per day during any trimester. Women were categorized as having used alcohol during pregnancy if they reported any alcohol use during any trimester. Women were asked when their prenatal care began (first, second or third trimester) and if they planned their pregnancy (yes/no). Maternal mood symptoms during pregnancy was defined as “present” if the mother answered positively to the following question: "During pregnancy, did you feel depressed or have any nervous condition?". Type of delivery was classified as vaginal or caesarean section.

The father's presence during child's life was reported by mothers at 24-month and 48-month follow-up and categorized as "never absent", "absent sometimes" and "always absent". Child variables were: sex; low birthweight (<2.500 g); gestational age (≤36, 37–41 or ≥42 weeks); 5 min Apgar score (< 7 or ≥ 7); duration of breastfeeding reported by mothers at the 24-month follow-up (< 1, 1–3, 3–6, 6–12 or ≥12 months); number of siblings older or younger than the cohort participant assessed at the 11-year follow-up (0, 1, 2, 3 or ≥ 4); Intelligence Quotient (IQ) assessed by the Wechsler Intelligence Scale for Children-III (WISC-III) at age six and categorized as high average (>0.75 SD), average (-0.75 to 0.75 SD) and low average (< -0.75 SD) (Wechsler, 1991).

### 2.5 Statistical analysis

First, we conducted a descriptive analysis of adolescents and mother's characteristics among those included and not included in the present analyses. All outcomes were analysed as continuous variables in original score units. Peer relationship and prosocial behaviour problems were also analysed as binary variables according to the clinical cut points previously explained. The mean score and standard deviation of the three outcomes according to maternal and child characteristics were analysed using ANOVA. Bivariate analyses were conducted to identify potential confounding variables. Multivariate linear regression analysis was conducted to evaluate the association between trajectories of maternal depression and changes in outcomes scores adjusting for selected confounding variables in separate models. Multivariate logistic regression analysis was used to estimate the association between the main exposure and dichotomized peer relationship and prosocial behaviour problems variables also controlling for selected confounding variables in separate models. Potential confounding variables were included as covariates if they were significantly associated with the main exposure (maternal depression trajectories) and outcome of interest and were not part of the causal chain (Rothman and Greenland, 1998. They were grouped and included in the adjusted analysis using a backward strategy selection (Victora et al., 1997). Five models were included for each outcome: unadjusted results (model 1), model 1 + socioeconomic variables (model 2), model 2 + maternal variables (model 3), model 3 + pregnancy and delivery variables (model 4) and model 4 + father's presence on child's life and child characteristics (model 5). If the significance level was below 0.20, the variable remained in the model as a potential confounder for the next level. All analyses were performed with Stata software version 14.2 (StataCorp LP, College Station, Tex).

The study was approved by the Research Committee of the University of São Paulo School of Medicine, (Research Protocol no. 402/18), and by the Research Ethics Committee of the Federal University of Pelotas. Written informed consent was obtained from the mothers or legal guardians of the adolescents. At the 11-year follow-up, adolescents also signed an informed consent form. Cases of severe mental health problems, as identified by the psychologists, were evaluated and, when necessary, were referred to the psychiatric or psychological care facilities available in the city.

## 3 Results

### 3.1 Attrition analysis

Of the 4231 participants constituting the original cohort, 98 died in the first 11 years of life and 3566 were interviewed at 11 years. Data about the outcomes (peer relationship problems, prosocial behaviour and locus of control at age 11) and main exposure were available for 3437 individuals (81.2% of the original cohort).Women who were included in the present study were more educated, were less likely to be single, to be in the lowest quintile of income, multiparous and to present mood symptoms during pregnancy. Also, the proportion of women that started prenatal care in the first semester of pregnancy was higher between those included than those non-included. Included and non-included women had similar age and did not differ significantly in the proportion of those who reported that had planned their pregnancies, smoked and consumed alcohol during pregnancy. In addition, adolescents included in the present analyses had higher birthweight, and lower frequencies of 5min Apgar score <7 and preterm birth than those not included (Table 1).

**Table 1:** Comparison of maternal and child characteristics between those included and not included in the present study, 2004 Pelotas Birth Cohort.

Variables	Included (*n* = 3437)	Not included (*n* = 794)	*p*-value*
Family income, lowest quintile (%)	19.1	26.9	**<0.001**
Schooling (years), mean (sd)	8.2 (3.4)	7.8 (3.7)	**0.005**⁎⁎
Maternal skin colour, white (%)	73.1	72.7	0.799
Maternal age (years), mean (sd)	26.2 (6.8)	25.5 (6.6)	0.103⁎⁎
Single mother (%)	15.5	20.4	**0.001**
Parity ≥2 (%)	33.3	38.6	**0.012**
Smoking during pregnancy (%)	26.8	30.2	0.053
Alcohol during pregnancy (%)	3.2	3.6	0.548
Started prenatal care in the 1st trimester (%)	74.1	67.4	**0.001**
Planned pregnancy (%)	43.8	41.6	0.257
Mood symptoms during pregnancy (%)	24.0	29.6	**0.001**
C-section (%)	45.0	47.0	0.330
Child sex, male (%)	51.2	54.8	0.071
Low birthweight (%)	8.3	17.6	**<0.001**
Preterm birth (<37 w) (%)	13.3	20.7	**<0.001**
5 min Apgar score, <7 (%)	1.40	5.6	**<0.001**

⁎ chi-square test.

⁎⁎ ANOVA test.

### 3.2 Maternal depression trajectories

Table 2 presents the EPDS scores mean and standard deviation in each follow-up wave.

**Table 2:** Description of maternal depressive symptoms through offspring's life and adolescent's socioemotional competences at age 11.

*Maternal depressive symptoms description*	
EPDS scores by follow-up waves^a^	
3 months, mean (SD)	6.25 (0.16)
12 months, mean (SD)	7.23 (0.08)
24 months, mean (SD)	7.37 (0.08)
48 months, mean (SD)	7.40 (0.09)
6 years, mean (SD)	7.09 (0.10)
11 years, mean (SD)	7.11 (0.09)

*Adolescent's outcomes description*	
Peer relationship problems, mean (SD)	1.36 (0.03)
% above clinical cut-off^b^	13.15
Prosocial behavior, mean (SD)	9.31 (0.02)
% above clinical cut-off^b^	1.28
Locus of control, mean (SD)	6.14 (0.03)

a Information on maternal depressive symptoms were available for *n* = 886 women (3 months), *n* = 3874 (12 months), *n* = 3821 (24 months), *n* = 3748 (48 months), *n* = 3302 (6 years), and *n* = 3517 women (11 years).

b Clinical range based on clinical cut-off of SDQ INFO, 2016.

Analysis of maternal depression trajectories showed that groups 1 ("low") and 2 ("moderate-low") including mothers with EPDS scores below 10 across all time points, thus suggesting low depressive symptomatology, comprised 74.8% of the mothers. Group 3 ("increasing") included 11.1% of the study women that had a consistent increase in depressive symptoms during the study period. The fourth group ("decreasing") containing 9.2% of the sample was composed by women that showed high EPDS scores in the first 2 years postpartum and a marked decrease afterwards. Group 5 ("high-chronic"), including mothers with high EPDS scores across the study period, comprised 4.9% of the sample (data not shown in the tables).

### 3.3 Factors associated with adolescents’ socioemotional scores

Table 2 presents the adolescents' socioemotional mean scores and the percentage of adolescents above the clinical cut-off.

Higher scores in peer relationship problems and lower prosocial behaviour scores were associated with most of the variables that indicate more socioeconomic and familiar disadvantages (Tables 3 and 4). Low birthweight, gestational age, 5min Apgar score and duration of breastfeeding were not associated with peer relationship problems score. Maternal skin colour, type of delivery, low birthweight and gestational age were not associated with prosocial behaviour score (Tables 3 and 4).

**Table 3:** Peer relationship problems, prosocial behaviour and locus of control scores according to family characteristics, 2004 Pelotas Birth Cohort.

Variables	N (%)	Peer relationship problems score Mean (SD)	*p*	Prosocial behaviour score Mean (SD)	*p*	Locus of control score Mean (SD)	*p*
***Family income (quintiles)***			**<0.001**		<0.001		<0.001
1 (lowest)	658 (19.1)	1.57 (1.73)		9.21 (1.33)		6.54 (1.96)	
2	692 (20.1)	1.57 (1.78)		9.30 (1.31)		6.53 (1.89)	
3	691 (20.1)	1.49 (1.85)		9.26 (1.42)		6.20 (1.95)	
4	734 (21.3)	1.22 (1.69)		9.38 (1.23)		5.87 (1.91)	
5 (highest)	662 (19.3)	0.98 (1.45)		9.37 (1.14)		5.60 (1.94)	
***Years of maternal schooling***			**<0.001**		**<0.001**		**<0.001**
0–4	504 (14.8)	1.68 (2.00)		9.19 (1.54)		6.71 (1.85)	
5–8	1411 (41.4)	1.52 (1.74)		9.30 (1.28)		6.33 (2.00)	
≥9	1492 (43.8)	1.12 (1.57)		9.35 (1.21)		5.80 (1.90)	
***Skin colour***			**0.010**		0.075		**<0.001**
White	2513 (73.1)	1.32 (1.71)		9.32 (1.27)		6.00 (1.97)	
Non-white	924 (26.9)	1.49 (1.75)		9.27 (1.34)		6.53 (1.90)	
***Living with a partner***			**0.002**		**<0.001**		**<0.001**
Yes	2904 (84.5)	1.58 (1.78)		9.33 (1.26)		6.09 (1.96)	
No	533 (15.5)	1.32 (1.71)		9.17 (1.44)		6.42 (1.96)	
***Age at childbirth (years)***			**0.002**		**0.009**		**0.041**
<20	651 (18.9)	1.58 (1.85)		9.18 (1.38)		6.30 (1.95)	
20–34	2309 (67.2)	1.32 (1.69)		9.32 (1.28)		6.12 (1.95)	
≥35	475 (13.8)	1.27 (1.68)		9.40 (1.22)		6.02 (2.04)	
***Parity***			**0.002**		**<0.001**		**<0.001**
0	1365 (39.7)	1.34 (1.71)		9.23 (1.33)		5.99 (1.94)	
1	926 (26.9)	1.64 (1.23)		9.41 (1.14)		6.08 (1.91)	
≥2	1146 (33.3)	1.49 (1.79)		9.30 (1.35)		6.38 (2.00)	
***Planned pregnancy***			**0.001**		**0.004**		**<0.001**
Yes	1504 (43.7)	1.25 (1.70)		9.35 (1.24)		5.99 (1.94)	
No	1932 (56.3)	1.45 (1.74)		9.27 (1.33)		6.26 (1.97)	
***Smoking during pregnancy***			**<0.001**		**<0.001**		**<0.001**
No	2515 (73.2)	1.23 (1.64)		9.34 (1.25)		6.03 (1.96)	
Yes	922 (26.8)	1.71 (1.89)		9.21 (1.38)		6.45 (1.93)	
***Alcohol during pregnancy***			**<0.001**		**0.006**		0.167
No	3326 (96.8)	1.34 (1.70)		9.32 (1.28)		6.15 (1.96)	
Yes	111 (3.2)	1.99 (2.18)		8.98 (1.53)		5.89 (1.99)	
***Depression during pregnancy***			**<0.001**		**<0.001**		**<0.001**
No	2610 (76.0)	1.23 (1.66)		9.35 (1.25)		6.06 (1.96)	
Yes	825 (24.0)	1.78 (1.86)		9.17 (1.41)		6.41 (1.96)	
***Started prenatal care visits***			**<0.001**		**<0.001**		**<0.001**
1st trimester	2519 (74.1)	1.28 (1.68)		9.34 (1.23)		5.99 (1.96)	
2nd trimester	794 (23.4)	1.54 (1.81)		9.22 (1.41)		6.55 (1.91)	
3rd trimester	84 (2.5)	1.80 (1.75)		9.18 (1.74)		6.59 (1.92)	
***Type of delivery***			**0.006**		0.564		**<0.001**
Vaginal	1888 (54.9)	1.44 (1.77)		9.29 (1.28)		6.29 (1.97)	
Caesarean section	1549 (45.1)	1.27 (1.67)		9.32 (1.30)		5.97 (1.95)	
***Father's presence during child's life (24–48 months)***			**0.001**		**<0.001**		**<0.001**
Never absent	2047 (66.8)	1.26 (1.67)		9.39 (1.18)		5.99 (1.98)	
Absent sometimes	581 (18.9)	1.44 (1.81)		9.20 (1.46)		6.28 (1.86)	
Always absent	437 (14.2)	1.56 (1.74)		9.20 (1.47)		6.47 (1.94)	
***Maternal depression trajectory***			**<0.001**		**<0.001**		**<0.001**
Low	1121 (32.6)	0.83 (1.33)		9.51 (1.05)		5.90 (1.95)	
Moderate low	1451 (42.2)	1.36 (1.67)		9.28 (1.30)		6.16 (1.95)	
Increasing	382 (11.1)	2.01 (1.94)		9.12 (1.41)		6.38 (1.94)	
Decreasing	315 (9.2)	1.83 (1.96)		9.11 (1.56)		6.43 (2.00)	
Chronic high	168 (4.9)	2.50 (2.20)		8.94 (1.61)		6.53 (1.96)

**Table 4:** Peer relationship problems, prosocial behaviour and locus of control scores according to child characteristics, 2004 Pelotas Birth Cohort.

Variables	N (%)	Peer relationship problems score Mean (SD)	*p*	Prosocial behaviour score Mean (SD)	*p*	Locus of control score Mean (SD)	*p*
***Sex***			**0.019**		**0.012**		0.059
Male	1761 (51.2)	1.43 (1.76)		9.24 (1.33)		6.08 (1.95)	
Female	1676 (48.8)	1.29 (1.68)		9.37 (1.25)		6.21 (1.98)	
***Low birthweight***			0.620		0.652		0.364
No	3153 (91.7)	1.36 (1.73)		9.31 (1.29)		6.13 (1.96)	
Yes	284 (8.3)	1.41 (1.61)		9.31 (1.31)		6.24 (1.99)	
***Gestational age***			0.056		0.095		0.146
≤36 weeks	456 (13.3)	1.43 (1.67)		9.22 (1.38)		6.26 (2.04)	
37–41 weeks	2761 (80.4)	1.33 (1.72)		9.32 (1.28)		6.11 (1.94)	
≥42 weeks	218 (6.3)	1.60 (1.90)		9.32 (1.30)		6.31 (2.04)	
***5 min. Apgar score***			0.054		**0.010**		0.592
≥7	3372 (98.6)	1.35 (1.72)		9.30 (1.30)		6.14 (1.96)	
<7	48 (1.4)	1.83 (1.94)		9.48 (0.97)		6.29 (1.97)	
***Duration of breastfeeding***			0.144		**<0.001**		0.816
Never	86 (2.5)	1.61 (1.90)		8.96 (1.82)		6.12 (1.97)	
<1 month	260 (7.6)	1.53 (1.91)		9.20 (1.37)		6.10 (1.97)	
1–3 months	521 (15.2)	1.45 (1.70)		9.37 (1.17)		6.24 (2.02)	
3–6 months	631 (18.4)	1.37 (1.72)		9.28 (1.34)		6.16 (2.00)	
6–12 months	614 (17.9)	1.28 (1.76)		9.34 (1.23)		6.08 (1.95)	
≥12 months	1321 (38.5)	1.31 (1.66)		9.32 (1.28)		6.13 (1.93)	
***Siblings (number)***			**<0.001**		**0.002**		<0.001
0	1275 (38.0)	1.29 (1.67)		9.25 (1.31)		5.97 (1.91)	
1	1203 (35.9)	1.29 (1.68)		9.36 (1.24)		6.04 (1.99)	
2	525 (15.6)	1.55 (1.82)		9.33 (1.27)		6.46 (2.01)	
3	189 (5.6)	1.61 (1.90)		9.29 (1.48)		6.63 (1.93)	
≥4	162 (4.8)	1.64 (1.85)		9.18 (1.44)		6.62 (1.81)	
***Intelligence quotient (IQ)***			**<0.001**		**<0.001**		**<0.001**
High average (>0.75 SD)	703 (21.6)	1.13 (1.62)		9.39 (1.21)		5.30 (1.77)	
Average (-0.75 to 0.75 SD)	1777 (54.7)	1.27 (1.65)		9.33 (1.25)		6.15 (1.90)	
Low average (< -0.75 SD)	769 (23.7)	1.77 (1.89)		9.22 (1.43)		6.88 (1.93)	

Higher external LoC scores were found in adolescents from mothers with the following characteristics: less educated; with low family income; with younger age; non-white; multiparous; single at childbirth; who reported mood symptoms during pregnancy; who reported smoking during pregnancy; who had an unplanned gestation; who started prenatal care later; and who had a vaginal delivery. Also, adolescents with more siblings, lower IQ scores and an absent father between age 2 and 4 showed an externality tendency on LoC (Tables 3 and 4).

Adolescents from mothers in the high-chronic depression trajectory had the lowest prosocial behaviour scores and the highest peer relationship problems and LoC scores than any other group (test for linear trend *p* < 0.001 for the three outcomes) (Table 3).

### 3.4 Adolescents’ outcomes at age 11 as a function of maternal depression trajectory membership

No sex interaction effect was observed for the association between maternal depression trajectory groups and peer relationship problems, prosocial behaviour and LoC scores (*p*-values = 0.394, 0.947 and 0.661, respectively). Thus, the analysis were conducted without stratification by adolescent's sex.

After fully adjustment, the changes in the scores for peer relationship problems and prosocial behaviour in all maternal depressive trajectories compared to the “low” group (reference category) did not substantially change for linear regression analysis. Mean peer relationship problems score was 1.37 points higher among children of mothers belonging to the high chronic trajectory when compared to those from mothers in the low trajectory group (reference) (Table 5). Mean prosocial behaviour score was 0.59 points lower among adolescents of mothers belonging to the high chronic trajectory when compared to those from mothers in the low trajectory group (reference) (Table 5). The same pattern of association was observed for logistic regression analysis with binary outcomes. The probability of adolescents presenting peer relationship and prosocial behaviour problems increased as we moved from the low to the high-chronic trajectory. In the fully adjusted analysis, adolescents from mothers in the high chronic trajectory were four and a half time more likely to have peer relationship 4.588 (OR 4.59, 95% CI 2.95–7.14) and prosocial behaviour problems (OR 4.511, 95% CI 1.299–15.668) when compared to adolescents of women in the low group (reference) (Table 6).

**Table 5:** Crude and adjusted analysis for socioemotional skills (peer relationship problems, prosocial behaviour and locus of control) according to the trajectories of maternal depressive symptoms (children from mothers in "low" group = reference), 2004 Pelotas Birth Cohort.

	Models	Maternal depression trajectories					
		Low	Moderate low	Increasing	Decreasing	Chronic high	*p*-value
		β (SE)	β (SE)	β (SE)	β (SE)	β (SE)	
Peer relationship problems	Model 1	1.000	0.530 (0.066)	1.178 (0.098)	0.996 (0.106)	1.670 (0.137)	**<0.001**
	Model 2^a^	1.000	0.488 (0.066)	1.074 (0.100)	0.888 (0.108)	1.557 (0.140)	**<0.001**
	Model 3^b^	1.000	0.488 (0.066)	1.074 (0.100)	0.888 (0.108)	1.557 (0.140)	**<0.001**
	Model 4^c^	1.000	0.448 (0.067)	0.997 (0.102)	0.791 (0.110)	1.386 (0.145)	**<0.001**
	Model 5^d^	1.000	0.430 (0.069)	0.965 (0.104)	0.800 (0.113)	1.371 (0.148)	**<0.001**
Prosocial behaviour	Model 1	1.000	–0.230 (0.051)	–0.386 (0.076)	–0.404 (0.082)	–0.571 (0.106)	**<0.001**
	Model 2^e^	1.000	–0.224 (0.051)	–0.379 (0.076)	–0.395 (0.081)	–0.568 (0.106)	**<0.001**
	Model 3^f^	1.000	–0.219 (0.051)	–0.371 (0.076)	–0.385 (0.082)	–0.569 (0.106)	**<0.001**
	Model 4^g^	1.000	–0.217 (0.051)	–0.370 (0.076)	–0.380 (0.082)	–0.549 (0.106)	**<0.001**
	Model 5^h^	1.000	–0.204 (0.053)	–0.329 (0.079)	–0.321 (0.087)	–0.595 (0.114)	**<0.001**
Locus of control	Model 1	1.000	0.261 (0.078)	0.480 (0.116)	0.533 (0.125)	0.633 (0.162)	**<0.001**
	Model 2^i^	1.000	0.164 (0.077)	0.211 (0.116)	0.276 (0.125)	0.312 (0.162)	**0.006**
	Model 3^j^	1.000	0.160 (0.077)	0.193 (0.116)	0.261 (0.124)	0.274 (0.162)	**0.012**
	Model 4^l^	1.000	0.128 (0.078)	0.129 (0.119)	0.227 (0.128)	0.169 (0.170)	0.081
	Model 5^d^	1.000	0.084 (0.081)	0.117 (0.125)	0.133 (0.139)	–0.018 (0.181)	0.499

Model 1 = crude.

Model 2 = model 1 + socioeconomic variables.

Model 3 = model 2 + maternal variables.

Model 4 = model 3 + pregnancy and delivery variables.

Model 5 = model 4 + paternal and child variables.

a Family income, maternal schooling, marital status.

b None variable remained in the model.

c Planned pregnancy, smoking during pregnancy, alcohol during pregnancy and mood symptoms during pregnancy.

d Child´s sex, father's presence in child's life during 24 to 48 months age, IQ.

e Marital status.

f Maternal age.

g Alcohol during pregnancy.

h Child´s sex, father's presence in child's life during 24 to 48 months age.

i Family income, maternal schooling, skin colour, marital status.

j Maternal age, parity.

l Smoking during pregnancy, alcohol during pregnancy, mood symptoms during pregnancy, started prenatal care, type of delivery.

**Table 6:** Crude and adjusted logistic regression analysis for SDQ socioemotional competences (peer relationship and prosocial behaviour problems) according to the trajectories of maternal depressive symptoms (children from mothers in "low" group = reference), 2004 Pelotas Birth Cohort.

	Models	Maternal depression trajectories					
		Low	Moderate low	Increasing	Decreasing	Chronic high	*p*-value
		OR (95%CI)	OR (95%CI)	OR (95%CI)	OR (95%CI)	OR (95%CI)	
Peer relationship problems	Model 1	1.000	1.972 (1.480–2.627)	4.556 (3.258–6.371)	3.862 (2.690–5.543)	6.713 (4.105–9.283)	**<0.001**
	Model 2^a^	1.000	1.828 (1.369–2.441)	3.829 (2.717–5.395)	3.264 (2.258–4.718)	5.026 (3.312–7.628)	**<0.001**
	Model 3^b^	1.000	1.837 (1.377–2.454)	3.823 (2.714–5.384)	3.235 (2.237–4.678)	5.097 (3.358–7.736)	**<0.001**
	Model 4^c^	1.000	1.823 (1.365–2.434)	3.809 (2.704–5.366)	3.187 (2.202–4.612)	4.844 (3.181–7.375)	**<0.001**
	Model 5^d^	1.000	1.772 (1.313–2.391)	3.590 (2.514–5.128)	3.354 (2.292–4.908)	4.588 (2.949–7.137)	**<0.001**
Prosocial behaviour problems	Model 1	1.000	2.466 (0.981–6.194)	2.464 (0.748–8.122)	5.466 (1.930–15.474)	5.700 (1.720–18.890)	**<0.001**
	Model 2^e^	1.000	2.298 (0.911–5.798)	1.994 (0.594–6.699)	4.508 (1.564–12.994)	4.191 (1.228–14.303)	**0.0438**
	Model 3^f^	1.000	2.298 (0.911–5.798)	1.994 (0.594–6.699)	4.508 (1.564–12.994)	4.191 (1.228–14.303)	**0.0438**
	Model 4^g^	1.000	2.178 (0.858–5.529)	2.045 (0.608–6.882)	4.514 (1.561–13.050)	4.149 (1.211 (14.211)	**0.0475**
	Model 5^h^	1.000	2.020 (0.783–5.209)	2.045 (0.603–6.942)	3.786 (1.229–11.663)	4.511 (1.299–15.668)	0.0601

Model 1 = crude.

Model 2 = model 1 + socioeconomic variables.

Model 3 = model 2 + maternal variables.

Model 4 = model 3 + pregnancy and delivery variables.

Model 5 = model 4 + paternal and child variables.

a Family income, maternal schooling.

b Maternal age.

c Alcohol during pregnancy.

d Child´s sex, 5min Apgar Score, IQ.

e Maternal schooling, marital status.

f None variable remained in the model.

g Trimester that started prenatal care.

h Father's presence in child's life during 24 to 48 months age.

After adjusting for pregnancy and delivery variables (model 4), the association between maternal depression trajectory groups and LoC scores was not significant. After fully adjustment, this association was also not statistically significant (*p* = 0.499) (Table 5).

## 4 Discussion

Using data from a population-based birth cohort study, we identified five distinct trajectories of maternal depressive symptoms from 3 months postpartum through the first 11 years of offspring´s life. Adolescents from mothers assigned to the “high-chronic’ depressive symptomatology showed the highest risk of presenting peer relationship problems and prosocial behaviour difficulties at age 11 compared to those whose mothers had low depressive symptoms, even after adjusting for socioeconomic, familiar and child characteristics. This association, however, was not observed for LoC scores after pregnancy and delivery variables were included in the adjusted models.

Our findings support previous studies that observed adverse socioemotional and behaviour outcomes among adolescents exposed to chronic maternal depression (Campbell et al., 2009, Korhonen et al., 2012, Murray et al., 2006, Sanger et al., 2015). In general, prolonged and severe maternal depressive symptoms predict adolescent's social and behavioural maladjustment, independently of the instrument used for outcome assessment. Campbell et al. (2009) indicated that chronic maternal depressive symptoms at varying levels of severity predict poorer offspring adjustment, compared to adolescents whose mothers reported low depressive symptomatology. Our results are in line with these results once adolescents from mothers belonging to any depressive symptoms trajectories groups (high chronic, increasing, decreasing or moderate low trajectories) presented higher levels of social and emotional problems when compared to those from mothers in the low depressive symptomatology trajectory group. For peer relationship problems scores, it was observed a slightly higher score among offspring from mothers on the increasing trajectory group compared to those from mothers belonging to the decreasing trajectory group. In other words, mothers in the increasing trajectory group presented high levels of depressive symptoms at the same time point as the outcome assessment of their offspring. This result evidences the adverse impact of current high maternal depressive symptoms on adolescent social skills.

Our study did not find sex interaction effect for any association between maternal depressive symptoms and offspring socioemotional competences. This result is contrary to earlier studies reporting that boys are more vulnerable to psychosocial development problems than girls when faced with maternal postnatal depression (Grace et al., 2003, Weinberg et al., 2006. Zahn-Waxler et al., 2006). Although the sex differences in socioemotional and psychopathology development are well established (Else-Quest et al., 2006, Zahn-Waxler et al., 2008), the sex interaction effect for the association between longitudinal maternal depressive symptoms and offspring mental health outcomes is not always reported in previous studies. In fact, to the best of our knowledge, we have not identified any article examining if maternal depression affects differently offspring peer relationship problems and prosocial behaviour according to adolescent's sex. Future research should explore and test sex-stratified models of risk to children of depressed mothers (Goodman et al., 2011).

Remarkably, our results suggest that maternal depressive symptoms during offspring's life is not a predictor of LoC orientation, opposed to what we have initially hypothesized. Although the antecedents of control expectancies in adolescence are not fully understood, previous studies have indicated that early experiences of adverse and uncontrollable circumstances may foster external LoC orientation characterized by diminished sense of perceived control over life events (Carton and Nowicki, 1994, Chorpita and Barlow, 1998, Nowicki and Duke, 2016). The exposure to parental depression and negative parenting styles were also associated to offspring external LoC (Chorpita and Barlow, 1998, Muris et al., 2004, Nowicki and Duke, 2016). Recent studies showed the mediating and moderating role that LoC orientation may exert between stress life events and unfavourable psychological outcomes (Culpin et al., 2015, Deardorff et al., 2003, Hunter et al., 2010, Kliewer and Sandier, 1992, Muris et al., 2004). Indeed, Culpin et al. (2015) reported that 34% of the total estimated association between early socioeconomic adversity and depression at age 18 was explained by external LoC at age 16. Thus, future studies should explore the mechanisms through which control beliefs mediate the association between disadvantageous factors in childhood and negative health outcomes in adult life.

The negative repercussion of maternal depression on offspring's socioemotional competences may be explained by several possible mechanisms. First of all, depressed mothers, especially those with more severe and recurrent symptoms, may transmit to their offspring genetic vulnerability to mental health problems (Silberg et al., 2010, Sullivan et al., 2000). Second, maternal depression often occurs within a context of environmental risk, which may also place children at increased risk for behaviour problems (Elgar et al., 2004). Indeed, Matijasevich et al. (2015), using the same cohort database of the present study, showed that maternal depressive symptoms severity and chronicity were associated with more adverse socioeconomic and contextual factors, such as lower familiar income, lower maternal schooling and unplanned pregnancy. All these factors together with potential familiar stressors (e.g. marital conflicts or partner violence) contribute to generate an accumulative negative context that may lead to offspring socioemotional maladjustment (Appleyard et al., 2005, Elgar et al., 2004). The early adolescence period represents a major transition stage of life, when youths are adjusting to a myriad of physical, cognitive and emotional changes within themselves while leading with social challenges (Crone and Dahl, 2012). The experience of living with a depressed mother may place adolescents at a particular risk for adjustment problems as a result of less engaged and supportive parenting and less effective role models (Campbell et al., 2009). Additionally, father's absence during children's life is not only related to the lack of spouse support and financial assistance that exacerbate maternal depressive symptom *per se*, but also eliminate the potential buffering effect of father involvement for children of depressed mothers. As showed by Collishaw et al. (2016), co-parent emotional support is a strong predictor of sustained good mental health and behavioural resilience in a sample of high-risk adolescents with a depressive parent. Finally, socioemotional development is an on-going and complex process that is affected by a range of biologic, environmental and social factors during individual's life (Wiggins and Monk, 2013). The impact of maternal depression on adolescent's socioemotional adjustment is presumed to have not only one particular pathway, but multiple mechanisms with reciprocal and interactional relationships between all involved factors (Goodman, 2007, Goodman and Gotlib, 1999).

Our study had a number of strengths, such as: (1) a large population-based sample with high response rate; (2) longitudinal assessments of maternal depression; (3) the use of validated instruments for assessing both maternal depressive symptomatology and adolescent's socioemotional competences; and (4) the adjustment for multiple maternal, familial, and child characteristics that potentially act as confounding variables for the associations being investigated. The study has also some limitations. As nearly 20% of original cohort were not included in the analysis due to missing data, selection bias may have been introduced in our study. However, we believe that the magnitude of association between maternal depressive symptoms and adolescent's socioemotional maladjustment was only marginally affected by sample attrition (Gustavson et al., 2012, Wolke et al., 2009). More importantly, the prevalence estimates of maternal depression and adolescent's outcomes were probably underestimated due to non-random non-response related to unfavourable socioeconomic factors (Lundberg et al., 2005, Wolke et al., 2009, Young et al., 2006). As an accurate estimation of mental health problems prevalence in the population is a key factor for service provision, this limitation should be considered in policy-making. Also, adolescent's socioemotional competences were ascertained by maternal report only, with exception of LoC, which was measured by child's self-report. As the 6-year follow-up used parent version of SDQ instrument, the same version was chosen for the 11-year follow-up aiming the comparability between the waves. Depressed mothers may be especially likely to report high levels of emotional and behavioural problems in their children (Muller and Furniss, 2013). However, previous studies have showed that depressed parents could be as accurate as other informants regarding their children behaviour (Lewis et al., 2012, Richters, 1992). Nevertheless, reports from multiple informants (e.g. fathers and teachers) or independent assessments may lead to more valid and precise measures of children's behaviour than maternal reports only (Dirks et al., 2012, Ordway, 2011) and should be considered in future research designs. As previously mentioned, father characteristics are a key factor for a number of adolescent outcomes. Unfortunately, data regarding paternal mental health, inter-parental conflicts, and other relevant aspects of paternal involvement in offspring's life were not available for the present study, evidencing another limitation. Finally, other limitation of our study is that even carefully designed longitudinal observational studies cannot unambiguously identify causal inferences, due to the possible role of other unmeasured confounders or reverse causation.

## 5 Conclusion

In conclusion, the present study strengthens the evidences of negative impact of severe and recurrent maternal depression on offspring's socioemotional competences. Aligned with previous studies that have demonstrated that persistent maternal depression affects emotional and behaviour development of children, our results extend this adverse impact to early adolescence, a period of life when youths are confronted with great social changes. Our study showed that adolescents from mothers not only on high-chronic but also on intermediated depressive symptoms trajectories presented more socioemotional difficulties than those of mothers with low depressive symptomatology. These findings highlight some important implications for mental health services: first, properly screening of women with recurrent elevated depressive symptoms at a subclinical level should be incorporated in clinical practice. Women presenting mild levels of depressive symptoms are often classified as not depressed and, as a consequence, they do not receive adequate assistance or treatment, when necessary. Second, the identification of maternal depressive symptomatology needs to be extended throughout offspring's life and not be focused only on pregnancy and early postpartum period. Further research should explore potential protective factors, such as behavioural resilience, for deleterious effects of maternal depression on offspring's socioemotional competences, as clearly not all mothers with depressive symptoms have offspring with adverse outcomes. Also, interventions that promote socioemotional competences development in children and adolescents may be an effective approach to prevent mental health problems in the future.

## Conflict of interest

The authors have no conflicts of interest to declare.

## Contributors

Jessica M. Maruyama undertook the analysis, interpreted the results and drafted the first version of the article. Alicia Matijasevich and Iná S. Santos participated in the design and analysis of the study and collaborated with the interpretation of the findings and writing of the article. Maria Pastor-Valero, Tiago N. Munhoz and Fernando C. Barros collaborated with the interpretation of the findings and writing of the article. All authors approved the final version of the manuscript submitted.

## Role of the funding source

This article is based on data from the 2004 Pelotas Birth Cohort study, conducted by the Postgraduate Program in Epidemiology of the Federal University of Pelotas, Brazil, with the support of the Brazilian Public Health Association (ABRASCO). From 2009 to 2013, the 2004 Pelotas birth cohort was supported by the Wellcome Trust (Grant no. 086974/Z/08/Z). Previous phases of the study were supported by the World Health Organization (Grant no. 03014HNI), National Support Program for Centres of Excellence (PRONEX) (Grant no. 04/0882.7), Brazilian National Research Council (CNPq) (Grant no. 481012-2009-5; 484077-2010-4; 470965-2010-0; 481141-2007-3), Brazilian Ministry of Health (Grant no. 25000.105293/2004-83) and Children's Pastorate. The 11-year follow-up was also funded by the São Paulo Research Foundation (FAPESP; grant no. 2014/13864-6). I.S.S., F.C.B and A.M. are supported by the CNPq.JM is supported by a grant from Sao Paulo Research Foundation (FAPESP; Research Grant no. 2017/22723-5). M.P.-V. was a Visiting Lecturer at the Department of Preventive Medicine, University of São Paulo, funded by the AIEF fellowship from the Miguel Hernández University.
